# Impact of Nutritional Status on Mortality in Older Patients Hospitalized for Acute Heart Failure

**DOI:** 10.3390/nu18040623

**Published:** 2026-02-13

**Authors:** Tsukasa Murakami, Keisuke Kojima, Masanori Takenoya, Kentaro Jujo, Ryusuke Ae, Masanari Kuwabara

**Affiliations:** 1 Department of Cardiology, Japanese Red Cross Ogawa Hospital, 1525 Ogawa, Ogawa-machi, Hiki, Saitama 355-0397, Japan; 2 Department of Cardiology, Saitama Medical Center, 1981 Kamoda, Kawagoe 330-8503, Japan; juken1123@mac.com; 3 Division of Cardiology, Department of Medicine, Nihon University School of Medicine, Tokyo 173-8610, Japan; 4 Division of Public Health, Center for Community Medicine, Jichi Medical University, 3311-1 Yakushiji, Shimotsuke 329-0498, Japan; shirouae@jichi.ac.jp; 5 Division of Cardiovascular Medicine, Department of Medicine, Jichi Medical University, 3311-1 Yakushiji, Shimotsuke 329-0498, Japan

**Keywords:** malnutrition, geriatric nutritional risk index, acute heart failure, older adults

## Abstract

**Backgrounds/Objectives:** Advances in prevention and medical care in the field of cardiology have led to an increase in the number of older patients with heart failure. In this population, assessment of nutritional status is particularly important. However, the prognostic impact of severity-based nutritional assessment at admission remains unclear. We conducted a study to elucidate the impact of malnutrition severity at admission on the prognosis of older patients hospitalized for acute heart failure (AHF). **Methods**: This study investigated the relationship between the Geriatric Nutritional Risk Index (GNRI) at admission and prognosis in 214 older patients aged ≥65 years who were hospitalized for AHF (mean age, 85 ± 8 years; male, 49%) between 2019 and 2023. GNRI was assessed by dividing patients into four groups: GNRI > 98 as normal (*n* = 64), 92 ≤ GNRI < 98 as mild risk (*n* = 54), 82 ≤ GNRI < 92 as moderate risk (*n* = 66), and GNRI < 82 as severe risk (*n* = 30). The discriminative performance of GNRI for 1-year all-cause mortality was compared with that of the Controlling Nutritional Status (CONUT) score and the Prognostic Nutritional Index (PNI). **Results**: During a median follow-up of 356 days, 76 deaths were observed. Worse GNRI categories were associated with older age, underweight, frailty, and anemia. Multivariable Cox proportional hazards models revealed that moderate GNRI risk (hazard ratio (HR), 2.69; 95% confidence interval (CI), 1.34–5.40) and severe GNRI risk (HR, 9.75; 95% CI, 4.30–22.10) were associated with higher all-cause mortality when compared with normal GNRI, along with age (HR per 1-year increase, 1.07; 95% CI, 1.03–1.11). Sensitivity analysis using GNRI as a continuous variable demonstrated similar results; GNRI was inversely associated with all-cause mortality (HR per 1 GNRI increase, 0.92; 95% CI, 0.90–0.95). In a subgroup analysis of age ≥85 years, the inverse association between GNRI and all-cause mortality was consistent. For 1-year all-cause mortality, GNRI showed moderate discrimination (area under the curve (AUC), 0.71; 95% CI, 0.63–0.80). Although the AUC of GNRI was not significantly different from that of the CONUT score or the PNI, GNRI demonstrated significantly better risk reclassification (net reclassification improvement, 0.47 vs. CONUT, *p* = 0.05; 0.43 vs. PNI, *p* = 0.02). **Conclusions**: In older patients with AHF including the oldest-old, nutritional status assessed by the GNRI at admission was predictive of prognosis. The importance of evaluating nutritional status at admission in clinical settings is reaffirmed.

## 1. Introduction

Advances in cardiovascular prevention and guideline-directed therapies, particularly improved reperfusion and pharmacological treatment for acute myocardial infarction, have substantially reduced early mortality, allowing many patients who would previously have died to survive into older age [1]. As a consequence, the number of elderly patients living with heart failure (HF) as a sequela of myocardial infarction has increased, and HF has become a major clinical and public health burden in aging societies [1,2]. Recent major guidelines highlight that this growing population of older HF patients requires optimized long-term management and implementation of evidence-based therapies [3,4]. Among HF conditions, acute heart failure (AHF) is a critical condition with a 1-year mortality of 11–27% [5,6,7,8,9], especially among older adults [8,9]. Given the high early mortality risk, timely identification of high-risk patients who may benefit from comprehensive management is critically important, especially in older adults with AHF.

Malnutrition is common among older adults with AHF and is associated with increased mortality [10,11,12,13,14,15,16,17,18]. AHF is associated with multiple pathophysiological mechanisms that adversely affect nutritional status, including reduced appetite, impaired nutrient absorption due to intestinal congestion, increased metabolic demand, and cytokine-mediated hypercatabolism [10]. These factors may contribute to progressive nutritional deterioration during the acute phase. In turn, malnutrition and AHF appear to interact bidirectionally, exacerbating systemic vulnerability and potentially contributing to poorer clinical outcomes. Various tools have been proposed to assess nutritional status in patients with HF [19,20]. Comprehensive instruments such as the Mini Nutritional Assessment are valuable for evaluating chronic nutritional status [21]; however, in older adults hospitalized with AHF, their assessment may be influenced by acute functional impairment, hemodynamic instability, and disease-related limitations. In this context, laboratory-based nutritional indices offer a more objective and timely approach to risk assessment.

The Geriatric Nutritional Risk Index (GNRI) is an objective tool used to assess the nutritional risk of older adults [22]. Malnutrition assessed by the GNRI shows an association with high mortality of patients with AHF [11,12,15,16,17,18]. Furthermore, prior comparative studies have suggested that GNRI is more strongly associated with all-cause mortality than other laboratory-based nutritional indices, such as the Prognostic Nutritional Index (PNI) and the Controlling Nutritional Status (CONUT) score, in patients with HF [23]. However, many studies dichotomized the study population by defining malnutrition as GNRI < 92 [11,15,16,17,18] or <98 [12]. As a result, the prognostic implications of malnutrition severity across the full spectrum of GNRI categories remain insufficiently clarified. GNRI was originally designed as a four-grade nutritional risk stratification system, rather than a binary indicator, suggesting that severity-based grading may provide more refined risk stratification. Although several studies have suggested the clinical relevance of GNRI severity categories in various cardiovascular populations [24,25,26], the impact of GNRI-based malnutrition severity has not been adequately investigated in patients with AHF. Moreover, evidence regarding the prognostic significance of GNRI in the oldest-old population (those aged 85 years or older), who are particularly vulnerable to malnutrition and adverse outcomes, remains limited. We therefore hypothesized that worse nutritional status at admission, assessed by severity-based GNRI classification, would be associated with higher mortality and could be useful in estimating risk in older adults with AHF. This study aimed to investigate the prognostic impact of malnutrition severity assessed by the GNRI on all-cause mortality of older adults with AHF.

## 2. Methods

This was an observational, single-center study. We reviewed consecutive patients with AHF who were admitted to the Japanese Red Cross Ogawa Hospital (JRCOH) (Saitama, Japan) between January 2019 and December 2023. JRCOH is a secondary emergency hospital located in an ageing area, where approximately 40% of residents are aged 65 years or older. We screened emergency hospitalized patients with diagnostic codes for HF in the International Classification of Diseases, 10th Revision (ICD-10) (I50.0, I50.1, I50.9, I11.0, I13.0, and I13.2) [27]. A total of 528 patients were initially identified. To ensure accurate case selection, we performed a detailed chart review of all these patients. Through this process, we excluded the following cases: patients who were not admitted on an emergency basis (*n* = 49), patients with cardiopulmonary arrest on arrival (*n* = 20), patients whose primary diagnosis was clearly not AHF (*n* = 17), and duplicate cases (*n* = 68). After these exclusions, 374 patients with AHF were finally identified. Among these patients, those aged <65 years and those whose GNRI could not be calculated at admission were further excluded. We acquired clinical information from hospital records. This study was approved by the institutional review board (approval number Ogawa-Rin-90, 12 March 2025) and was conducted in accordance with the Declaration of Helsinki. Since this was a retrospective study without additional interventions, the opt-out method was used.

The GNRI is an objective tool created to predict malnutrition-related complications in older adults [22]. The GNRI was calculated as follows: 14.89 × serum albumin level (g/dL) + 41.7 × (body weight (kg)/ideal body weight (kg)) [22]. When the body weight-to-ideal body weight ratio was greater than 1, the value was set to 1, as in previous studies [22,28]. The ideal body weight was calculated by the Lorentz formula as follows: ideal body weight for men (kg) = height (cm) − 100 − (height (cm) − 150)/4 and for women (kg) = height (cm) − 100 − (height (cm) − 150)/2.5 [22,28]. GNRI > 98 was classified as normal, GNRI of 92 to 98 as mild nutritional risk, GNRI of 82 to <92 as moderate nutritional risk, and GNRI < 82 as severe nutritional risk, respectively [22]. The CONUT score was calculated by summing up designated points of three laboratory markers (serum albumin, total lymphocyte counts, and total cholesterol). The CONUT score ranges from 0 to 12 [29]. The PNI was calculated as follows: 10 × serum albumin in g/dL + 0.005 × total lymphocyte count in 1 μL [30]. Frailty was evaluated using the Clinical Frailty Scale (CFS) [31]. According to the CFS, patient frailty before admission was scored as follows: (i) very fit; (ii) well; (iii) managing well; (iv) vulnerable; (v) mildly frail; (vi) moderately frail; (vii) severely frail; (viii) very severely frail; and (ix) terminally ill [31]. Patients with CFS ≥ 4 were defined as frail [31]. Using echocardiography, left ventricular ejection fraction (LVEF) and left-sided valvular diseases were examined. Significant valve disease was defined as worse than moderate in severity. The primary outcome was all-cause mortality.

### Statistical Analysis

Data are presented as percentages for categorical variables, mean ± standard deviation for normally distributed continuous variables, or median and interquartile range for non-normally distributed continuous variables. Statistical significance was set at *p* < 0.05. The study population was divided into four GNRI categories: normal, mild risk, moderate risk, and severe risk based on GNRI at admission. First, baseline characteristics among the four groups were compared. Categorical variables were compared using Fisher’s exact test. Normally distributed continuous variables were compared using one-way ANOVA. Otherwise, continuous variables were compared using the Kruskal–Wallis test. The cumulative incidence of all-cause mortality was compared using the Kaplan-Meier method. Then, we performed multivariable Cox proportional hazards models to calculate the hazard ratio (HR) for GNRI for all-cause mortality. Considering the small number of events and missing data in BNP levels (missing in 14% of patients), we performed three multivariable analyses as follows. In model 1, GNRI category was adjusted for age, sex, hypertension, diabetes mellitus, dyslipidemia, estimated glomerular filtration rate (GFR) < 60 mL/min/1.73 m^2^, and New York Heart Association (NYHA) class. In model 2, pre-admission history of HF hospitalization, history of percutaneous coronary intervention (PCI) or coronary artery bypass grafting (CABG), cancer, and hemoglobin levels were further adjusted. In model 3, log BNP was additionally adjusted, and the other variables were the same as in model 2. To calculate the HR, normal GNRI was used as the reference.

As a primary sensitivity analysis, GNRI as a continuous metric was tested in these three models. As a secondary sensitivity analysis, to address potential overfitting in the more complex models, we performed stepwise variable selection. Specifically, stepwise Cox proportional hazards models with backward elimination based on the likelihood ratio test were applied to the covariates included in models 2 and 3 (entry criterion *p* = 0.05; removal criterion *p* = 0.10). Moreover, a subgroup analysis according to age, sex, body mass index (BMI), NYHA class, frailty, anemia, and presence of peripheral edema was performed. Anemia was defined as a hemoglobin level <13.0 g/dL in men and <12.0 g/dL in women according to the criteria defined by the World Health Organization [32]. In the subgroup analysis, the HR of the GNRI (as a continuous metric) for all-cause mortality was calculated after adjustment for age and sex.

We also performed an exploratory analysis evaluating GNRI at discharge. After excluding in-hospital deaths, patients with available discharge GNRI were analyzed to investigate temporal changes in nutritional status based on GNRI during hospitalization.

For 1-year all-cause mortality, the discriminative performance of GNRI was evaluated using receiver operating characteristic (ROC) curve analysis, and its area under the curve (AUC) was compared with those of the CONUT score and the PNI using DeLong’s test. Net reclassification improvement (NRI) was calculated to assess whether GNRI provided better risk reclassification than the CONUT score and the PNI.

SPSS ver. 20 for Windows (SPSS, Inc., Chicago, IL, USA) was used for the description of demographic data, survival analysis, and multivariable analysis. To evaluate the association between GNRI and all-cause mortality, the curve was estimated using restricted cubic splines in R v4.2.1 (R Foundation for Statistical Computing, Vienna, Austria), and packages of “rms (version 6.4-1)”, “survival (version 3.3-1)”, “dplyr (version 1.1.0)”, and “ggplot2 (version 3.5.2)” were used.

## 3. Results

**Figure 1:**
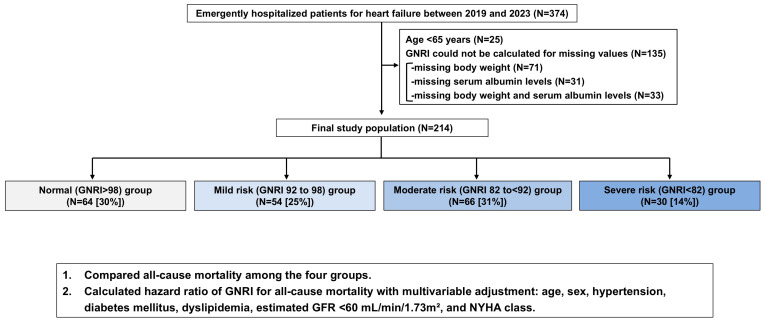
Study flow chart. Abbreviations: GNRI, Geriatric Nutritional Risk Index; GFR, glomerular filtration rate; NYHA, New York Heart Association.

During the study period, 374 patients were admitted on an emergency basis for AHF. Of these, 25 patients aged <65 years and 135 patients whose GNRI could not be calculated at admission (71 patients without recorded body weight, 31 patients without serum albumin levels, and 33 patients without both variables) were excluded, resulting in a final study population of 214 patients (Figure 1).

The mean age of the final study population was 85 ± 8 years (49% male). Patients were divided into four GNRI risk groups: normal (*n* = 64, 30%), mild risk (*n* = 54, 25%), moderate risk (*n* = 66, 31%), and severe risk (*n* = 30, 14%). Patients with higher nutritional risk were older (82 ± 8, 86 ± 8, 86 ± 7, and 85 ± 8 years, in normal, mild-risk, moderate-risk, and severe-risk groups, respectively, *p* = 0.01), had lower BMI (25.0 ± 3.9, 23.7 ± 3.2, 20.9 ± 3.2, and 19.1 ± 3.7, *p* < 0.01), hemoglobin levels (12.4 ± 2.3, 11.1 ± 2.0, 10.7 ± 2.5, and 10.8 ± 1.8, *p* < 0.01), and albumin levels (4.1 ± 0.3, 3.6 ± 0.2, 3.3 ± 0.2, and 2.7 ± 0.4, *p* < 0.01), and had a higher prevalence of CFS ≥ 4 (40%, 56%, 60%, and 70%, *p* = 0.03) and peripheral edema (64%, 78%, 82%, and 90%, *p* = 0.03) (Table 1). Regarding nutritional assessment tools, CONUT and PNI showed consistent results with GNRI. Patients with worse GNRI had longer length of hospital stay (15 [11–24], 17 [13–33], 24 [15–47], and 27 [11–52], *p* = 0.01) and higher prevalence of in-hospital death (5%, 4%, 17%, and 40%, *p* < 0.01).

**Figure 2:**
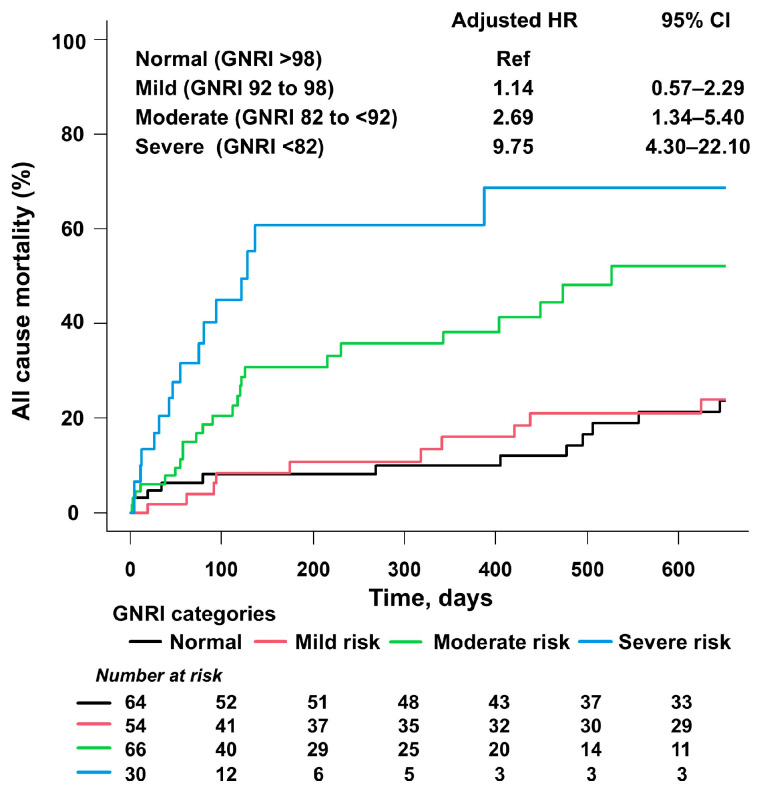
GNRI risk categories and all-cause mortality. Moderate and severe GNRI risk were associated with higher rates of all-cause mortality after multivariable adjustment. Adjusted HRs for GNRI risk were calculated using a multivariable Cox proportional hazards analysis with adjustment for age, sex, hypertension, diabetes mellitus, dyslipidemia, estimated GFR < 60 mL/min/1.73 m^2^, and NYHA class. The normal GNRI category was used as the reference. Abbreviations: GNRI, Geriatric Nutritional Risk Index; HR, hazard ratio; CI, confidence interval; GFR, glomerular filtration rate; NYHA, New York Heart Association.

During a median follow-up of 356 days (interquartile range, 66–919 days), 76 all-cause deaths were observed. The cumulative incidence of all-cause mortality was the highest in the severe-risk group (53%), followed by those in the moderate-risk group (36%), the mild-risk group (33%), and the normal group (28%) (*p* < 0.01) (Figure 2).

**Figure 3:**
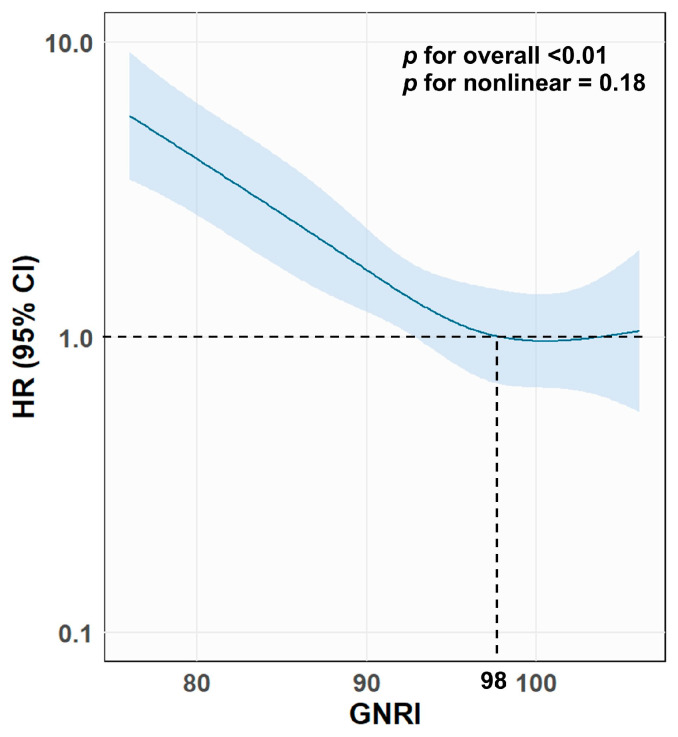
GNRI as a continuous variable and all-cause mortality. The association between GNRI and the HR for all-cause mortality was demonstrated using a restricted spline curve (knot = 4, reference = GNRI 98). Age and sex were adjusted to calculate HR. Abbreviations: GNRI, Geriatric Nutritional Risk Index; HR, hazard ratio.

Similarly, cumulative 1-year mortality was the highest in the severe-risk group (50%), followed by those in the moderate-risk group (30%), the mild-risk group (13%), and the normal group (9%) (*p* < 0.01). After adjustment for age and sex, a restricted spline curve demonstrated an inverse association between GNRI (as a continuous metric) and all-cause mortality (*p* for overall association < 0.01, *p* for non-linearity = 0.18) (Figure 3).

Multivariable Cox proportional hazards models showed that moderate risk (HR, 2.69; 95% confidence interval (CI) 1.34–5.40; *p* = 0.01) and severe risk (HR, 9.75; 95% CI, 4.30–22.10; *p* < 0.01), and age (HR per 1-year increase, 1.07; 95% CI, 1.03–1.11; *p* < 0.01) were associated with all-cause mortality after multivariable adjustment in model 1 (Table 2). Mild risk (HR, 1.14; 95% CI, 0.57–2.29; *p* = 0.71) was not significantly different from normal GNRI.

**Figure 4:**
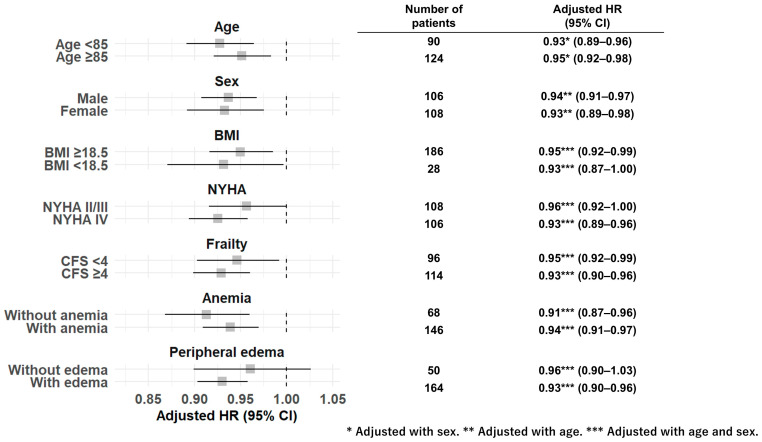
Subgroup analysis. GNRI (as a continuous variable) showed a consistent association with all-cause mortality regardless of age ≥ 85, sex, BMI < 18.5, NYHA class IV, CFS ≥ 4, and anemia. HRs were calculated with adjustment for age and sex. Abbreviations: HR, hazard ratio; BMI, body mass index; NYHA, New York Heart Association; CFS, Clinical Frailty Scale.

In model 2, moderate risk (HR, 2.61; 95% CI, 1.26–5.38; *p* = 0.01) and severe risk (HR, 9.62; 95% CI, 4.20–22.02; *p* < 0.01) remained significant after further adjustment. However, in model 3, severe risk (HR, 8.12; 95% CI, 3.07–21.48; *p* < 0.01) remained statistically significant after adjustment for log BNP; conversely, moderate risk was not statistically significant (Table 3). The primary sensitivity analysis, in which GNRI was treated as a continuous variable, revealed that GNRI was inversely associated with all-cause mortality after multivariable adjustment as in model 1 (HR per 1 increment, 0.92; 95% CI, 0.90–0.95; *p* < 0.01) (Table 2). In addition, the GNRI as a continuous variable was consistently associated with all-cause mortality after further adjustments, as in models 2 and 3 (Table 3). Regarding the secondary sensitivity analysis, in model 2 after stepwise selection, age (HR per 1-year increase, 1.08; 95% CI, 1.04–1.12; *p* < 0.01), prior heart failure admission (HR, 2.00; 95% CI, 1.24–3.25; *p* = 0.01), moderate GNRI risk (HR, 2.09; 95% CI, 1.08–4.02; *p* = 0.03), and severe GNRI risk (HR, 7.33; 95% CI, 3.45–15.59; *p* < 0.01) were retained in the final model. In model 3 after stepwise selection, age (HR per 1-year increase, 1.09; 95% CI, 1.05–1.13; *p* < 0.01), prior heart failure admission (HR, 2.08; 95% CI, 1.20–3.61; *p* = 0.01), log BNP (HR, 2.89; 95% CI, 1.49–5.61; *p* < 0.01), and GNRI severe risk (HR, 7.27; 95% CI, 2.96–17.85; *p* < 0.01) were retained, whereas moderate GNRI risk (HR, 1.70; 95% CI, 0.80–3.61; *p* = 0.17) was not. When GNRI was analyzed as a continuous variable, GNRI remained independently associated with all-cause mortality in both stepwise model 2 (HR per 1 GNRI increase, 0.93; 95% CI, 0.91–0.96; *p* < 0.01) and model 3 (HR per 1 GNRI increase, 0.94; 95% CI, 0.91–0.97; *p* < 0.01). Moreover, the GNRI (as a continuous metric) showed a consistent association with all-cause mortality regardless of age ≥ 85, sex, BMI < 18.5, NYHA class IV, CFS ≥ 4, and anemia (Figure 4). The GNRI was also associated with all-cause mortality in patients with edema. Although not statistically significant, a numerical trend for higher mortality with lower GNRI was observed in patients without edema.

Regarding temporal changes in GNRI during hospitalization, after excluding 28 in-hospital deaths, discharge GNRI was available in 104 of 186 patients (56%). GNRI significantly decreased from admission to discharge (92.6 ± 7.8 vs. 87.5 ± 9.5; *p* < 0.01). During a median follow-up of 460 days (interquartile range, 80–1083 days) from discharge, 25 all-cause deaths were observed. In univariable Cox proportional hazards models, lower admission GNRI (as a continuous variable) showed only a marginal association with all-cause mortality (HR 0.95, 95% CI, 0.90–1.01, *p* = 0.07), whereas lower discharge GNRI was significantly associated with increased mortality (HR 0.91, 95% CI, 0.87–0.95, *p* < 0.01). In multivariable Cox proportional hazards models, admission GNRI was not associated with all-cause mortality (HR 0.96, 95% CI, 0.91–1.01, *p* = 0.14); however, lower discharge GNRI remained significant after adjustment for age and sex (HR 0.92, 95% CI, 0.88–0.96, *p* < 0.01).

Regarding the discriminative performance of nutritional indices for 1-year all-cause mortality, among the 214 patients with available GNRI at admission, the AUC for GNRI was 0.71 (95% CI, 0.63–0.80). Among the 99 patients with available CONUT at admission, the AUC for GNRI was 0.66 (95% CI, 0.52–0.81), whereas the AUC for CONUT was 0.63 (95% CI, 0.50–0.77), with no significant difference between the two indices (*p* = 0.64). However, GNRI demonstrated significantly improved risk reclassification compared with CONUT, with an NRI of 0.47 (*p* = 0.05). Similarly, among the 169 patients with available PNI at admission, the AUC for GNRI was 0.73 (95% CI, 0.64–0.83), and that for PNI was 0.70 (95% CI, 0.60–0.79). The difference in AUC did not reach statistical significance (*p* = 0.17). Nevertheless, GNRI showed a significantly better reclassification ability than PNI, with an NRI of 0.43 (*p* = 0.02).

## 4. Discussion

This study revealed that nutritional status assessed by GNRI at admission was predictive of prognosis in older patients with AHF, with a mean age of 85 years. A clear and graded association between GNRI-based malnutrition severity and mortality risk was detected, with an 8% decrease in mortality risk per 1-point increase in GNRI, even in the oldest-old (those aged 85 years or older). Moreover, the moderate and severe GNRI risk categories were independently associated with 2.7-fold and 9.8-fold higher HRs of all-cause mortality compared with the normal GNRI risk category, respectively. By evaluating not only the presence of malnutrition but also its severity, mortality risk in older adults with AHF can be estimated more accurately. Importantly, our results underscore the clinical relevance of nutritional assessment at the time of hospital admission for early prognostic evaluation in this high-risk population.

### 4.1. Severe Malnutrition in Older Adults with AHF

In older adults with AHF, we revealed a high prevalence of malnutrition at admission, reaching 70% for at least mild nutritional risk (GNRI < 98) and 45% for worse than moderate nutritional risk (GNRI < 92). Malnutrition is common in older adults with heart diseases, such as atrial fibrillation [33], coronary artery disease [34], valvular heart disease [35,36], HF with preserved ejection fraction [28], and AHF [11,12,13,14,15,16,17,18]. Although the prevalence of malnutrition depends on the study population and the used assessment tool, AHF is one of the heart diseases in which malnutrition is frequently observed [11,12,13]. According to previous reports from Japan (mean age, 77–79 years), the prevalence of malnutrition (defined as GNRI < 92) was 33–48% in patients with AHF [11,15,16,17]. This was comparable to our result (45%). However, in this study, we also demonstrated that 14% of patients had severe nutritional risk (GNRI < 82) at admission. Although the prevalence of severe malnutrition in AHF has been unclear, our results might provide insight into the importance of severe malnutrition in the oldest-old population. Aging plays a key role in the development of malnutrition [10,37]. Considering the aging trend of patients with AHF [7], the prevalence of severe malnutrition in patients with AHF will increase, and malnutrition in AHF will be a more critical concern. Furthermore, we showed a worsening trend in frailty as nutritional risk increased. In patients with HF, a complex interplay of neurohormonal derangement, inflammation, appetite suppression, malabsorption, and decreased mobility contributes to the development of malnutrition and muscle wasting [10,38]. These wasting conditions likely act as mutual aggravators, contributing to poor clinical outcomes. Therefore, in older adults with AHF, coexisting frailty should be assessed when malnutrition is suspected.

### 4.2. The Prognostic Impact of Increasing Malnutrition Severity

In the present study, we demonstrated a graded increase in mortality risk with worsening GNRI-based nutritional status in older adults hospitalized with AHF. Previous studies and meta-analyses have consistently shown that malnutrition is associated with adverse outcomes in patients with AHF [11,12,15,16,17,18,19,39]. However, most prior investigations primarily focused on the presence or absence of malnutrition using binary GNRI cutoff values, such as <92 or <98, and therefore provided limited insight into the prognostic implications of malnutrition severity. The GNRI was originally developed as a four-grade nutritional risk stratification tool [22], suggesting that severity-based classification may enable more refined risk assessment than conventional dichotomous approaches. Although several studies have suggested the clinical relevance of GNRI severity categories in various cardiovascular populations, including patients with chronic heart failure [24] and those with valvular heart disease [25,26], data specifically evaluating GNRI-based malnutrition severity—particularly severe malnutrition defined as GNRI < 82—in patients with AHF remain scarce. Nakamura et al. reported that GNRI < 92 was associated with all-cause mortality in patients aged 80 years or older with AHF, representing one of the oldest study populations to date (mean age: 87 years) [18]. Nevertheless, similar to most previous studies [11,12,15,16,17], nutritional status was dichotomized, and the prognostic impact of increasing malnutrition severity was not fully explored. Oldest-old patients with AHF constitute a particularly high-risk population, characterized by a high prevalence of malnutrition, frequent comorbidities, and poor short-term outcomes. In such patients, early risk stratification at the time of hospital admission is clinically crucial, given the substantial risk of in-hospital mortality. Yoshihisa et al. previously demonstrated that severe GNRI-based nutritional risk was associated with poor prognosis in patients with AHF; however, their analysis was based on GNRI assessed at discharge, and the study population was relatively younger (mean age: 67 years) [40]. In contrast, the prognostic value of admission GNRI-based severity grading in the oldest-old patients with AHF has remained unclear.

By applying the original severity-based GNRI classification at admission, our study demonstrated that both moderate (GNRI 82 to <92) and severe (GNRI < 82) nutritional risk categories were independently associated with increased all-cause mortality, with a clear stepwise increase in risk as GNRI worsened. These associations remained consistent after multivariable adjustment and across subgroup analyses accounting for comorbidities and vulnerability-related conditions common in older adults with AHF. Taken together, these findings underscore the importance of evaluating not only the presence of malnutrition but also its severity to appropriately estimate mortality risk in this high-risk population. Our results further suggest that admission GNRI–based severity grading provides clinically meaningful early prognostic information in the oldest-old patients (those aged 85 years or older), a population that has been underrepresented in previous studies.

### 4.3. Clinical Implications

We demonstrated considerable 1-year mortality after AHF in patients with moderate GNRI risk (33%) and severe GNRI risk (50%) that was much higher than previously reported rates (11–27%) [5,6,7,8,9]. Importantly, these patients exhibited a high burden of in-hospital adverse outcomes, characterized by markedly elevated in-hospital mortality (17% in moderate risk and 40% in severe risk) and a prolonged length of hospital stay, reflecting greater clinical instability and care complexity during the acute phase. As we demonstrated, worse nutritional status was associated with vulnerable conditions, including older age, underweight, frailty, and anemia. These findings suggest that the GNRI could enable early screening of high-risk, vulnerable patients who require comprehensive care soon after admission. In addition to optimization of guideline-directed medical therapy for HF, support for coexisting comorbidities might contribute to better outcomes in malnourished patients with AHF. Although our additional analysis of discharge GNRI was limited by sample size, it suggested that GNRI may deteriorate during hospitalization and that GNRI assessed at discharge may better reflect post-discharge prognosis, consistent with previous observations by Ono et al. [41]. Furthermore, Kitagawa et al. reported that longitudinal worsening of GNRI is associated with increased mortality in patients with HF [42]. While it remains unclear whether GNRI-guided management directly improves prognosis, these findings collectively suggest that serial assessment of GNRI may allow more accurate and timely risk estimation during the clinical course. Regarding nutritional intervention in AHF, randomized controlled trials remain limited; however, several trials have demonstrated that nutritional interventions can improve mortality, quality of life, and readmission outcomes in patients with HF [43,44]. In addition, as shown in our study and others [45], malnutrition and frailty frequently coexist. Given that comprehensive cardiac rehabilitation programs incorporating nutritional support have been associated with improvements in nutritional status and physical function in elderly cardiovascular populations [46], integration of nutritional assessment with early rehabilitation may be clinically relevant as part of a multidisciplinary approach for older adults with AHF. From a practical perspective, an admission GNRI assessment could be used to trigger early, multidisciplinary nutritional management—such as prompt referral to a dietitian, individualized energy and protein goals, and coordination with rehabilitation to prevent further functional decline—especially in patients classified as having moderate-to-severe nutritional risk.

### 4.4. Limitations

This study has several limitations. First, since this was a retrospective, single-center study, there could be a selection bias. However, this study, conducted in a rural area in Japan, was able to follow an aging population until the occurrence of outcomes. Second, a substantial number of patients were excluded due to missing data required for GNRI calculation, most commonly missing body weight at presentation, as routine weight measurements is frequently challenging in very elderly and clinically severe patients. This may have introduced selection bias, as excluded patients were older, had a higher prevalence of NYHA class IV, and had lower serum albumin levels, suggesting poorer nutritional status. In addition, excluded patients exhibited a higher risk of all-cause death. Consequently, the prognostic impact of malnutrition observed in this study may have been underestimated. Nevertheless, the association between malnutrition and all-cause mortality remained consistent after multivariable adjustment and stratified analyses, supporting the robustness of the main findings. Third, although GNRI was calculated at admission, volume congestion in AHF might influence the assessment of nutritional status based on GNRI. Nevertheless, we demonstrated the usefulness of GNRI as a prognostic marker in older adults with AHF. Subgroup analyses showed a consistent association between GNRI and mortality in patients with peripheral edema, which may support this interpretation. Fourth, given the limited number of events relative to the number of covariates, multivariable models 2 and 3 might be susceptible to overfitting. To address this issue, we performed additional sensitivity analyses using stepwise variable selection. Although GNRI remained associated with mortality in these stepwise-selected models, results derived from more complex models should be interpreted with caution. Fifth, the number of patients in the severe GNRI risk group was relatively small (*n* = 30), which may limit the precision and stability of the estimated hazard ratios. However, the consistent dose-response association between GNRI and mortality observed in spline analyses and sensitivity analyses using GNRI as a continuous variable might support the robustness of the overall relationship. Finally, the present study focused on GNRI as the primary nutritional index. Our findings suggest that GNRI may be useful for prognostic risk estimation in older adults with AHF, including comparisons with PNI and CONUT; however, these comparative analyses were performed in limited patient subsets because of missing data and should be interpreted with caution. Further prospective studies are warranted to validate our findings.

## 5. Conclusions

Moderate and severe GNRI risk categories and lower GNRI at admission were independently associated with all-cause mortality in older adults with AHF. Malnutrition severity assessed by GNRI may be useful for estimating mortality risk among older adults with AHF, even in patients aged 85 years or older.

## Figures and Tables

**Table 1:** Clinical characteristics.

	Normal (N = 64)	Mild Risk (N = 54)	Moderate Risk (N = 66)	Severe Risk (N = 30)	*p*-Value
Age, years	82 ± 8	86 ± 8	86 ± 7	85 ± 8	0.01
Female, *n* (%)	20 (31)	37 (69)	39 (59)	12 (40)	<0.01
Height (cm)	157 ± 10	151 ± 8	153 ± 9	154 ± 8	<0.01
Body weight (kg)	62 ± 12	54 ± 10	49 ± 9	45 ± 8	<0.01
BMI (kg/m^2^)	25.0 ± 3.9	23.7 ± 3.2	20.9 ± 3.2	19.1 ± 3.7	<0.01
NYHA class, *n* (%)					0.18
II	9 (14)	4 (7)	5 (8)	1 (3)	
III	30 (47)	22 (41)	29 (44)	8 (27)	
IV	25 (39)	28 (52)	32 (49)	21 (70)	
Comorbidities					
Hypertension, *n* (%)	55 (86)	47 (87)	47 (71)	25 (83)	0.11
Diabetes mellitus, *n* (%)	21 (33)	12 (22)	18 (27)	13 (43)	0.21
Dyslipidemia, *n* (%)	20 (31)	9 (17)	11 (17)	5 (17)	0.15
COPD, *n* (%)	9 (14)	3 (6)	6 (9)	6 (20)	0.18
Dementia, *n* (%)	10 (16)	9 (17)	12 (18)	8 (27)	0.61
History of cancer, *n* (%)	13 (20)	10 (19)	13 (20)	8 (27)	0.83
Prior hospitalization due to HF, *n* (%)	18 (28)	22 (41)	27 (41)	10 (33)	0.39
Atrial fibrillation, *n* (%)	35 (55)	27 (50)	27 (41)	9 (30)	0.11
History of PCI or CABG, *n* (%)	7 (11)	4 (7)	12 (18)	4 (13)	0.36
PAD, *n* (%)	2 (3)	3 (6)	1 (2)	3 (10)	0.24
Living alone, *n* (%)	16/63 (25)	10/54 (19)	9/66 (14)	4/30 (13)	0.35
Clinical frailty scale ≥ 4, *n* (%)	25/63 (40)	29/52 (56)	39/65 (60)	21/30 (70)	0.03
Weight loss in the prior 3 months, *n* (%)	3/62 (5)	0/54 (0)	11/66 (17)	3/30 (10)	<0.01
Medication					
ACE-inhibitor, ARB, *n* (%)	32/64 (50)	24/53 (45)	22/66 (33)	9/30 (30)	0.13
β-blocker, *n* (%)	22/64 (34)	15/53 (28)	17/66 (26)	9/30 (30)	0.76
MRA, *n* (%)	11/64 (17)	9/53 (17)	13/66 (20)	6/30 (20)	0.96
SGLT-2 inhibitor, *n* (%)	4/64 (6)	3/53 (6)	4/66 (6)	2/30 (7)	1.00
Diuretics, *n* (%)	29/64 (45)	29/53 (55)	36/66 (55)	12/30 (40)	0.43
Vital signs					
Systolic blood pressure (mmHg)	139 ± 23	138 ± 24	136 ± 27	139 ± 27	0.92
Diastolic blood pressure (mmHg)	81 ± 19	81 ± 19	77 ± 15	76 ± 19	0.38
Heart rate (bpm)	86 ± 24	91 ± 24	85 ± 21	87 ± 18	0.47
Findings of congestion					
Pulmonary congestion or pleural effusion in chest imaging, *n* (%)	58/64 (91)	50/54 (93)	64/65 (99)	27/29 (93)	0.21
Peripheral edema, *n* (%)	41 (64)	42 (78)	54 (82)	27 (90)	0.03
Laboratory data					
Hemoglobin (g/dL)	12.4 ± 2.3	11.1 ± 2.0	10.7 ± 2.5	10.8 ± 1.8	<0.01
BUN (mg/dL) (*n* = 213)	26 ± 15	27 ± 14	29 ± 17	27 ± 16	0.63
Creatinine (mg/dL)	1.3 ± 0.8	1.2 ± 0.6	1.2 ± 0.8	1.1 ± 0.5	0.61
Estimated GFR (mL/min/1.73 m^2^)	48 ± 20	46 ± 20	49 ± 26	59 ± 30	0.12
Sodium (mEq/L)	140 ± 4	139 ± 6	138 ± 6	139 ± 6	0.03
BNP (pg/mL) (*n* = 183)	520 (274–834)	622 (295–1051)	777 (489–1508)	315 (167–1114)	0.01
Albumin (g/dL)	4.1 ± 0.3	3.6 ± 0.2	3.3 ± 0.2	2.7 ± 0.4	<0.01
GNRI	102.2 ± 3.9	94.9 ± 1.6	87.4 ± 2.7	75.7 ± 5.5	<0.01
CONUT (*n* = 99)	3 (2–4)	3 (2–5)	5 (3–6)	7 (7–8)	<0.01
PNI (*n* = 169)	47.9 ± 6.0	41.7 ± 3.6	38.0 ± 2.9	31.3 ± 4.3	<0.01
Echocardiography					
LVEF (%) (*n* = 195)	50 ± 16	53 ± 16	51 ± 19	55 ± 15	0.49
Significant AS, *n* (%)	2/60 (3)	4/50 (8)	4/60 (7)	0/25 (0)	0.50
Significant AR, *n* (%)	15/60 (25)	9/50 (18)	13/60 (22)	2/25 (8)	0.33
Significant MR, *n* (%)	30/60 (50)	19/50 (38)	27/60 (45)	6/25 (24)	0.14
In-hospital outcomes					
Length of hospital stay, days	15 (11–24)	17 (13–33)	24 (15–47)	27 (11–52)	0.01
In-hospital deaths, *n* (%)	3 (5)	2 (4)	11 (17)	12 (40)	<0.01

Data are expressed as median and interquartile range or number (percentage). Abbreviations: BMI; body mass index, NYHA, New York Heart Association; COPD, chronic obstructive pulmonary disease; HF, heart failure; PCI, percutaneous coronary intervention; CABG, coronary artery bypass grafting; PAD, peripheral artery disease; ACE, angiotensin-converting enzyme; ARB, angiotensin II receptor blocker; MRA, mineralocorticoid receptor antagonist; SGLT, sodium-glucose cotransporter; BUN, blood urea nitrogen; GFR, glomerular filtration rate; BNP, brain natriuretic peptide; GNRI, Geriatric Nutritional Risk Index; CONUT, Controlling Nutritional Status; PNI, Prognostic Nutritional Index; LVEF, left ventricular ejection fraction; AS, aortic stenosis; AR, aortic regurgitation; MR, mitral regurgitation.

**Table 2:** Univariable and multivariable Cox proportional hazards models to investigate the association of GNRI with all-cause mortality.

	Crude			Adjustment (GNRI Category)			Adjustment (Continuous GNRI)		
	HR	95% CI	*p*-Value	HR	95% CI	*p*-Value	HR	95% CI	*p*-Value
Age	1.08	1.04–1.12	<0.01	1.07	1.03–1.11	<0.01	1.06	1.02–1.10	<0.01
Female	0.90	0.57–1.41	0.65	0.81	0.51–1.28	0.36	0.80	0.51–1.27	0.35
Hypertension	1.30	0.68–2.46	0.43	1.50	0.75–3.03	0.25	1.57	0.79–3.11	0.19
Diabetes mellitus	0.53	0.31–0.93	0.03	0.72	0.38–1.34	0.16	0.73	0.39–1.36	0.32
Dyslipidemia	0.46	0.24–0.88	0.02	0.60	0.30–1.20	0.22	0.67	0.34–1.34	0.26
NYHA class									
II	Ref			Ref			Ref		
III	0.68	0.33–1.38	0.28	0.74	0.35–1.57	0.30	0.70	0.33–1.47	0.35
IV	1.00	0.51–1.97	1.00	0.90	0.43–1.86	0.77	0.78	0.38–1.60	0.49
Estimated GFR < 60 mL/min/1.73 m^2^	1.37	0.82–2.28	0.23	1.70	0.99–2.95	0.06	1.52	0.89–2.61	0.13
GNRI category									
Normal	Ref			Ref					
Mild risk	1.44	0.74–2.79	0.28	1.14	0.57–2.29	0.71			
Moderate risk	2.75	1.45–5.21	<0.01	2.69	1.34–5.40	0.01			
Severe risk	6.47	3.12–13.40	<0.01	9.75	4.30–22.10	<0.01			
GNRI (per 1 increment)	0.94	0.92–0.96	<0.01				0.92	0.90–0.95	<0.01

In this model, GNRI category and GNRI as a continuous variable were adjusted for age, sex, hypertension, diabetes mellitus, dyslipidemia, estimated GFR < 60 mL/min/1.73 m^2^, and NYHA class. Abbreviations: GNRI, Geriatric Nutritional Risk Index; HR, hazard ratio; CI, confidence interval; NYHA, New York Heart Association; GFR, glomerular filtration rate.

**Table 3:** Multivariable Cox proportional hazards models to investigate the association of GNRI with all-cause mortality.

	Model 2						Model 3					
	GNRI Category			Continuous GNRI			GNRI Category			Continuous GNRI		
	HR	95% CI	*p*-Value	HR	95% CI	*p*-Value	HR	95% CI	*p*-Value	HR	95% CI	*p*-Value
Age	1.07	1.02–1.11	<0.01	1.06	1.02–1.10	<0.01	1.08	1.03–1.13	<0.01	1.07	1.02–1.11	<0.01
Female	0.83	0.50–1.37	0.47	0.78	0.47–1.29	0.33	0.77	0.43–1.38	0.38	0.78	0.43–1.40	0.40
Hypertension	1.31	0.65–2.66	0.45	1.37	0.69–2.74	0.37	1.29	0.50–3.37	0.60	1.44	0.57–3.66	0.45
Diabetes mellitus	0.74	0.39–1.42	0.37	0.75	0.39–1.43	0.38	0.75	0.36–1.57	0.45	0.79	0.38–1.62	0.51
Dyslipidemia	0.67	0.33–1.37	0.27	0.76	0.37–1.55	0.45	0.83	0.36–1.91	0.66	0.85	0.38–1.91	0.69
NYHA class												
II	Ref			Ref			Ref			Ref		
III	0.71	0.33–1.52	0.38	0.67	0.31–1.45	0.31	0.64	0.27–1.50	0.30	0.59	0.25–1.41	0.24
IV	0.97	0.45–2.09	0.94	0.85	0.40–1.81	0.67	1.06	0.45–2.51	0.90	0.91	0.39–2.14	0.83
Estimated GFR < 60 mL/min/1.73 m^2^	1.55	0.88–2.71	0.13	1.43	0.82–2.52	0.21	1.28	0.66–2.47	0.47	1.15	0.60–2.22	0.67
Prior heart failure admission	2.00	1.15–3.50	0.01	1.80	1.05–3.10	0.03	2.18	1.13–4.21	0.02	1.87	1.00–3.51	0.05
History of PCI or CABG	0.62	0.28–1.41	0.26	0.62	0.28–1.35	0.23	0.67	0.27–1.65	0.39	0.70	0.30–1.67	0.42
Cancer	0.88	0.48–1.61	0.67	0.76	0.41–1.39	0.37	1.23	0.61–2.47	0.57	1.05	0.53–2.12	0.88
Hemoglobin	1.01	0.89–1.14	0.88	1.01	0.90–1.14	0.87	1.01	0.87–1.18	0.87	1.02	0.88–1.17	0.84
Log BNP							3.12	1.50–6.48	<0.01	2.63	1.29–5.37	0.01
GNRI category												
Normal	Ref						Ref					
Mild risk	0.97	0.47–1.98	0.93				0.94	0.41–2.13	0.87			
Moderate risk	2.61	1.26–5.38	0.01				1.86	0.80–4.30	0.15			
Severe risk	9.62	4.20–22.02	<0.01				8.12	3.07–21.48	<0.01			
GNRI per 1 increment				0.92	0.89–0.95	<0.01				0.93	0.90–0.97	<0.01

In model 2, GNRI was adjusted for age, sex, hypertension, diabetes mellitus, dyslipidemia, estimated GFR < 60 mL/min/1.73 m^2^, NYHA class, prior heart failure admission, history of PCI or CABG, cancer, and hemoglobin. In model 3, log BNP was added to model 2. Abbreviations: GNRI, Geriatric Nutritional Risk Index; HR, hazard ratio; CI, confidence interval; NYHA, New York Heart Association; GFR, glomerular filtration rate; PCI, percutaneous coronary intervention; CABG, coronary artery bypass grafting; BNP; brain natriuretic peptide.

## Data Availability

The original contributions presented in this study are included in the article. Further inquiries can be directed to the first author (T.M.). T.M. had full access to all of the data and takes responsibility for the integrity of the data and the accuracy of the data analysis.

## Abbreviations

HF = heart failure; AHF = acute heart failure; GNRI = Geriatric Nutritional Risk Index; JRCOH = the Japanese Red Cross Ogawa Hospital; ICD-10 = International Classification of Diseases, 10th Revision; PNI= Prognostic Nutritional Index; CFS = Clinical Frailty Scale; LVEF = left ventricular ejection fraction; GFR = glomerular filtration rate; NYHA = New York Heart Association; PCI = percutaneous coronary intervention; CABG = coronary artery bypass grafting; BMI = body mass index; COPD = chronic obstructive pulmonary disease; PAD = peripheral artery disease; ACE = angiotensin-converting enzyme; ARB = angiotensin II receptor blocker; MRA = mineralocorticoid receptor antagonist; SGLT = sodium-glucose cotransporter; BUN = blood urea nitrogen; BNP = brain natriuretic peptide; AS = aortic stenosis; AR = aortic regurgitation; MR = mitral regurgitation.
